# “Prostate telocytes change their phenotype in response to castration or testosterone replacement”

**DOI:** 10.1038/s41598-019-40465-1

**Published:** 2019-03-06

**Authors:** Sérgio Luis Felisbino, Bruno Domingos Azevedo Sanches, Flávia Karina Delella, Wellerson Rodrigo Scarano, Fernanda Cristina Alcântara Dos Santos, Patrícia Simone Leite Vilamaior, Sebastião Roberto Taboga, Luis Antônio Justulin

**Affiliations:** 1 Sao Paulo State University - UNESP, Institute of Biosciences, Laboratory of Extracellular Matrix, Prof. Dr. Antônio Celso Wagner Zanin St., 250, Rubião Júnior District, Botucatu, São Paulo 18618-689 Brazil; 2 Department of Structural and Functional Biology, State University of Campinas, Bertrand Russel Av., Campinas, São Paulo Brazil; 3 Department of Histology, Embryology and Cell Biology, Federal University of Goiás, Samambaia II, Goiânia, Goiás 74001970 Brazil; 4 Sao Paulo State University - UNESP, Department of Biology, Laboratory of Microscopy and Microanalysis, Cristóvão Colombo St., 2265 São José do Rio Preto, São Paulo Brazil

## Abstract

Telocytes are CD34-positive cells with a fusiform cell body and long, thin cytoplasmic projections called telopodes. These cells were detected in the stroma of various organs, including the prostate. The prostate is a complex gland capable of undergoing involution due to low testosterone levels; and this condition can be reversed with testosterone replacement. Telocyte function in the mature prostate remains to be dermined, and it is not known whether telocytes can take place in tissue remodeling during prostate involution and regrowth. The present study employed structural, ultrastructural and immunohistochemical methods to investigate the telocyte’s phenotypes in the ventral prostate (VP) from control (CT), castrated (CS) and testosterone replacement (TR) groups of adult male Wistar rats. Telocytes were found in the subepithelial, perimuscular and interstitical regions around glandular acini. Telocytes from CT animals have condensed chromatin and long and thin telopodes. In CS group, telocytes appeared quiescent and exhibited layers of folded up telopodes. After TR, telocytes presented loose chromatin, abundant rough endoplasmic reticulum and enlarged telopodes, closely associated with bundles of collagen fibrils. We called these cells “telocytes with a synthetic phenotype”. As testosterone levels and glandular morphology returned toward to the CT group parameters, after 10 days of TR, these telocytes progressively switched to the normal phenotype. Our results demonstrate that telocytes exhibit phenotypic plasticity upon androgen manipulation and interact with fibroblast and smooth muscle cells to maintain glandular architecture in control animals and during tissue remodeling after hormonal manipulation.

## Introduction

A few decades ago, researchers thought the stroma was a static environment that only act to support and nourish the epithelia. As such, the cellular milieu would be dominated by fibroblasts that would alternate between a more active state, the activated fibroblasts, whose function was restricted to the production of components of the extracellular matrix, and a less active state, the quiescent fibroblasts. However, there is now abundant knowledge that fibroblasts comprise a heterogeneous population of cells that may have different origins and functions in stromal maintenance^1–3^, and even quiescent fibroblasts are no longer seen as static cells since they demonstrate high metabolic activity^4^. In addition to fibroblast population heterogeneity in normal tissues, demonstrated mainly in the dermis, fibroblasts exhibit great plasticity in pathological conditions, such as cancer-associated fibroblasts^5^ and as part of myofibroblasts^6,7^. This diversity adds complexity to the study of the stroma. Fibroblast morphology may also change in specific contexts such as hypoxia^8^, a finding that indicates that these cells exhibit a high level of phenotypic plasticity. The same alteration was verified in smooth muscle cells, which exhibit phenotypic plasticity with the alternation between contractile and synthetic phenotypes, the latter of which synthesises elements of the extracellular matrix during musculature development during certain physiological or pathological contexts^9,10^. Additionally, in pathological conditions, there may be a transition from a contractile to a migratory and phagocytic phenotype^11^.

The stroma complexity has increased with the classification of a new cell type, the telocytes. Telocytes are CD34- or CD34- and c-Kit-positive cells that differ morphologically from fibroblasts, pericytes and smooth muscle cells since they have fine and long cytoplasmic extensions called telopodes. These structures are divided into dilated sections, podoms (with mitochondria), and fibrillar-like sections, the podomers that display a moniliform aspect^12^. Telocytes were detected in the myocardium^13,14^, skeletal muscle^15^, jejunum^16,17^, mammary gland^18^, joints^19^, sclera^20^, prostate^21,22^ and several other organs. The exact function of this cell type remains unclear, although there is evidence that telocytes exert organ-specific functions involved in homeostasis, remodelling, regeneration, repair, embryogenesis, angiogenesis and others^23^. The intimate association of telocytes with smooth muscle cells has been observed in several organs^13,22,24,25^. Indeed, it was proposed that telocytes play a supportive role in the prostate gland, including smooth muscle cell differentiation and contribution to tissue organisation during development^26^, but the function of telocytes in the mature prostate remains elusive.

Prostate physiology depends on steroid hormones, concentrations of which change throughout life. In some species, the prostate gland undergoes drastic seasonal changes^27,28^. In humans, the prostate responds to increases in testosterone (T) concentration, which can lead to pathological conditions such as hyperplasia^29,30^. Further, T deprivation or castration lead to prostate size reduction and hyperplasia reversion^28,31^. Antiandrogen therapies act to reduce the prostatic volume 15 to 25% through apoptosis and glandular epithelial compartment shrinkage^32^. In rodents, castration also reduced prostate volume and increased apoptosis of luminal epithelial cells^33^, while T replacement induced epithelial cell proliferation and restored the secretory epithelium activity^34,35^. In addition to changes in epithelial cells, androgen deprivation promotes smooth muscle cell transition from the contractile to the synthetic phenotype, without affecting its differentiation status^36,37^. Telocytes are closely associated with smooth muscle in the prostate^21,22^ and they play an important role in prostate tissue organisation during its initial development^26^, but little is known about telocyte behaviour in the context of androgen deprivation or T replacement. Thus, the present study uses histochemical, immunohistochemical and ultrastructural techniques to investigate whether telocytes are affected by castration and T replacement in the prostate and whether they play a role in prostate gland involution reversal due to T replacement.

## Results

### Light microscopy

**Figure 1:**
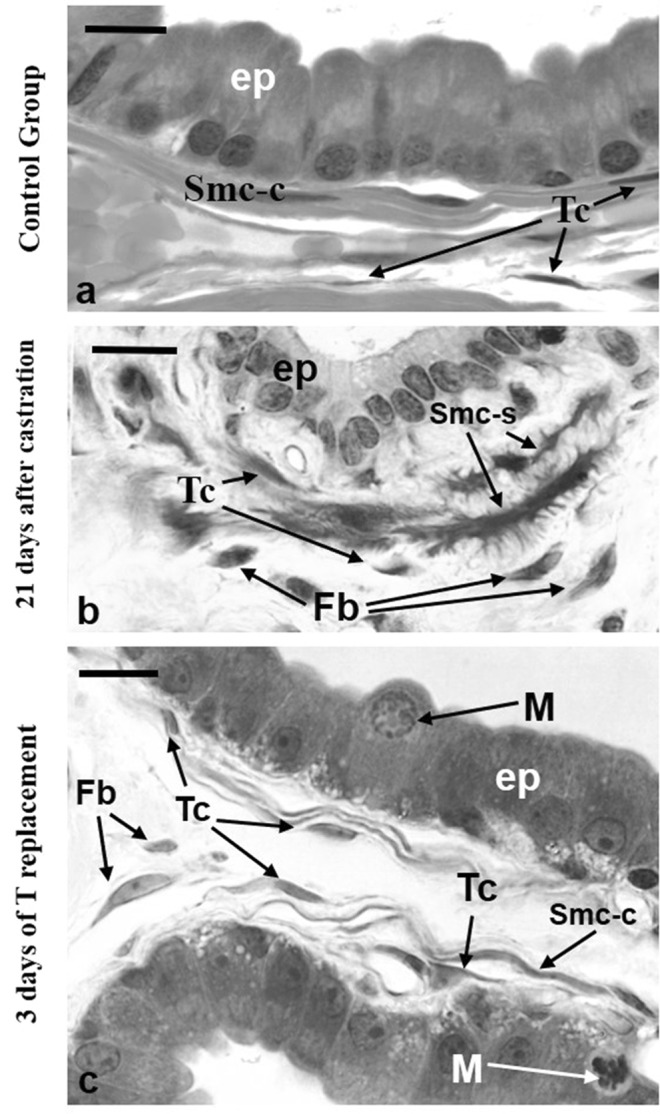
Resin-embedded sections of rat ventral prostates from the three different experimental group of animals: control, castrated and castrated plus testosterone (T) replacement. (**a**) In the control group, possible telocytes were observed in the subepithelial and perimuscular regions. These cells had spindle cell bodies and a reduced cytoplasm. Fibroblasts were also observed in the interstitial region. (**b**) Twenty-one days after castration, telocytes appeared with reduced or absent telopodes and enlarged cell bodies. Smooth muscle cells displayed irregular outline or with “spinous cell shape”. (**c**) After three days of T replacement, smooth muscle cells progressively returned to their contractile phenotype, possible telocytes with abundant cytoplasm, dilated nuclei or still incipient telopodes were observed in the subepithelial and perimuscular region, and fibroblasts were observed in the interalveolar region. Abbreviations: Ep (epithelium); Tc (telocytes), smc-c (smooth muscle cells with contractile phenotype); smc-s (smooth muscle cells with synthetic phenotype); Fb (fibroblasts); M (mitosis). Scale bar is 10 μm.

In the CT group, light microscopy indicated possible telocytes or telocytes-like cells located in the sub-epithelial region and the perimuscular region of rat VP. These cells had spindle cell bodies, reduced cytoplasm and possible long and thin telopodes (Fig. 1a). Twenty-one days after castration, these cells presented small telopodes and enlarged cell bodies, while smooth muscle cells appeared with irregular outline or with “spinous cell shape” (Fig. 1b). After 3 days of T replacement, smooth muscle cells returned to their regular outline, while telocytes-like exhibited abundant cytoplasm and long telopodes located in the subepithelial and perimuscular region. Fibroblasts were observed in the interstitial region (Fig. 1c).

### Ultrastructural analysis

**Figure 2:**
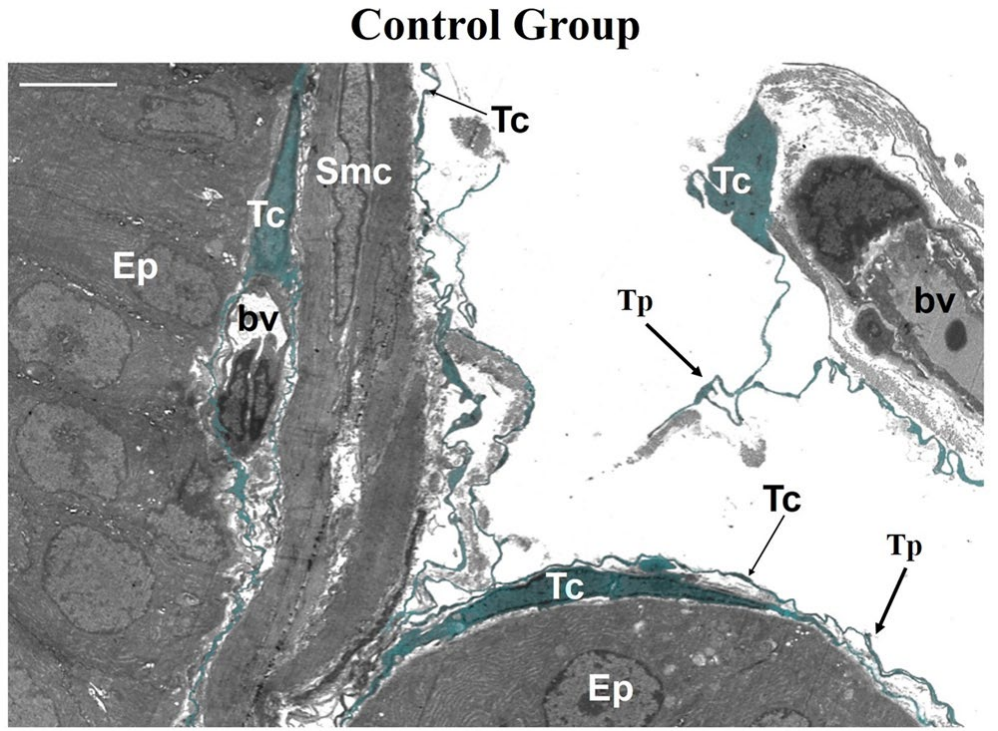
Ultrastructure of the ventral prostate of control male rats. Telocytes were observed at three sites: subepithelial, close to the glandular epithelial basement membrane; perimuscular, involving the smooth muscle cell layer; and interstitial, distributed in the interstitium in close association with thickened collagen fibres and blood vessels. Telocytes exhibited condensed chromatin and long, flattened cytoplasmic processes (telopodes). Abbreviations: Tc (telocytes); Tp (telopodes); Ep (epithelium); Smc (smooth muscle cells); bv (blood vessel). Scale bar is 5 μm.

**Figure 3:**
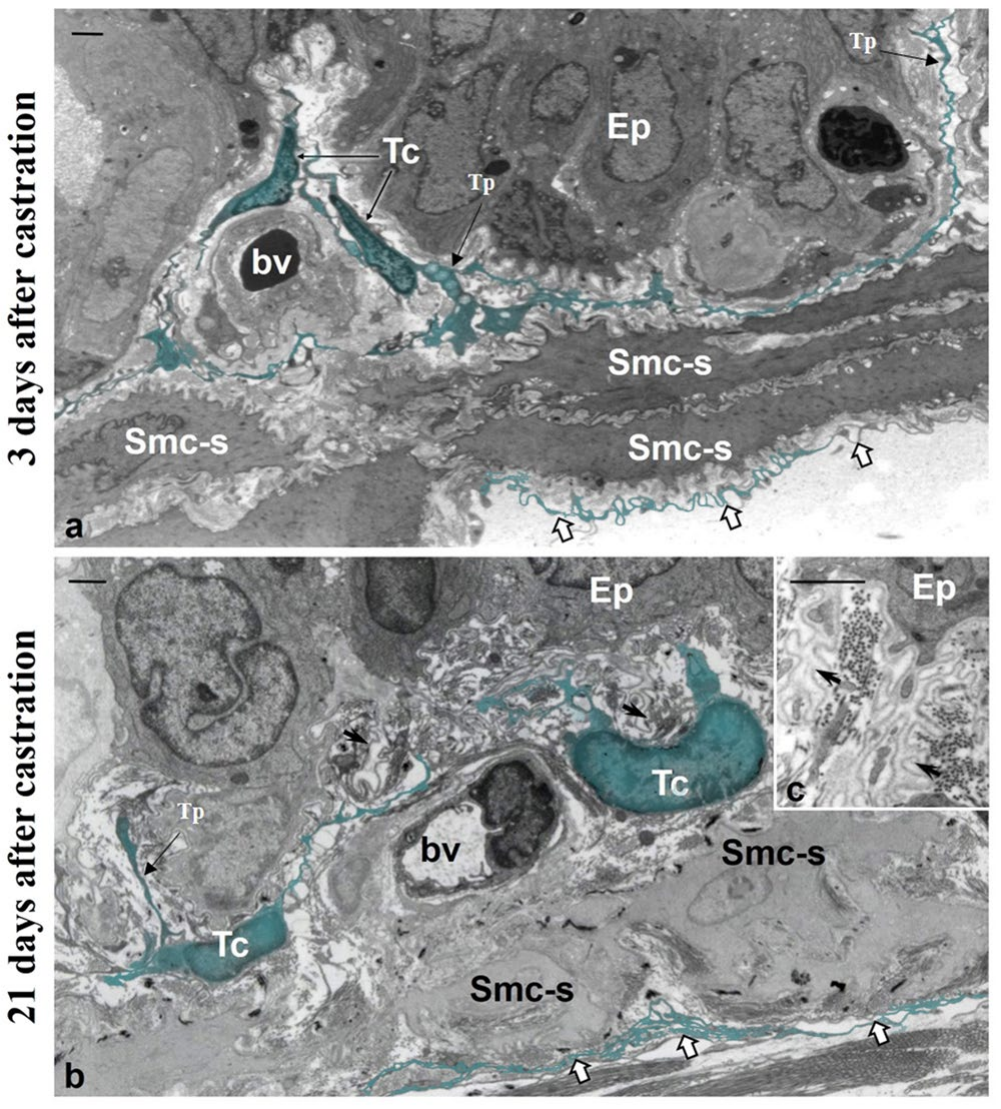
Ventral prostate ultrastructure of animals on day 3 and day 21 after castration. (**a**) In the castrated animal, prostatic telocytes exhibit dilated telopodes, whereas smooth muscle cells already have an irregular outline and evident nucleoli, indicating the transition for the synthetic phenotype. (**b**,**c**) Twenty-one days after castration, subepithelial telocytes present partial or total loss of their telopodes, while the perimuscular telocytes exhibit folded telopodes. Smc-s (smooth muscle cells with synthetic phenotype) Tc (Telocyte), Tp (Telopodes); Ep (Epithelium); bv (Blood vessel). Scale bar (1 μm).

Telocyte characteristics are best observed using transmission electron microscopy. In the VP from CT rats, telocytes were found at three sites: subepithelial, close to the glandular epithelial basement membrane; perimuscular, involving the smooth muscle cell layer; and interstitial, distributed in the interstitium in close association with thickened collagen fibres and blood vessels. In the CT group, telocytes exhibited condensed chromatin and long and thin telopodes (Fig. 2). We verified that telocytes underwent morphological changes upon androgen deprivation. Three days after castration, telopodes were dilated and retracted. Smooth muscle cells also underwent morphological changes, including an irregular outline and evident nucleoli, findings that indicate a transition from contractile to synthetic phenotype. Further, the prostatic epithelium also changed, namely the epithelial basement membrane presented numerous folds (Fig. 3a). Twenty-one days after castration, the epithelial basement membrane appeared highly folded and without connections with epithelial cells. Perimuscular telocytes have folded telopodes, while subepithelial telocytes show partial or total loss of their telopodes. These have also condensed chromatin, which indicates a quiescent or atrophic phenotype (Fig. 3b,c).

**Figure 4:**
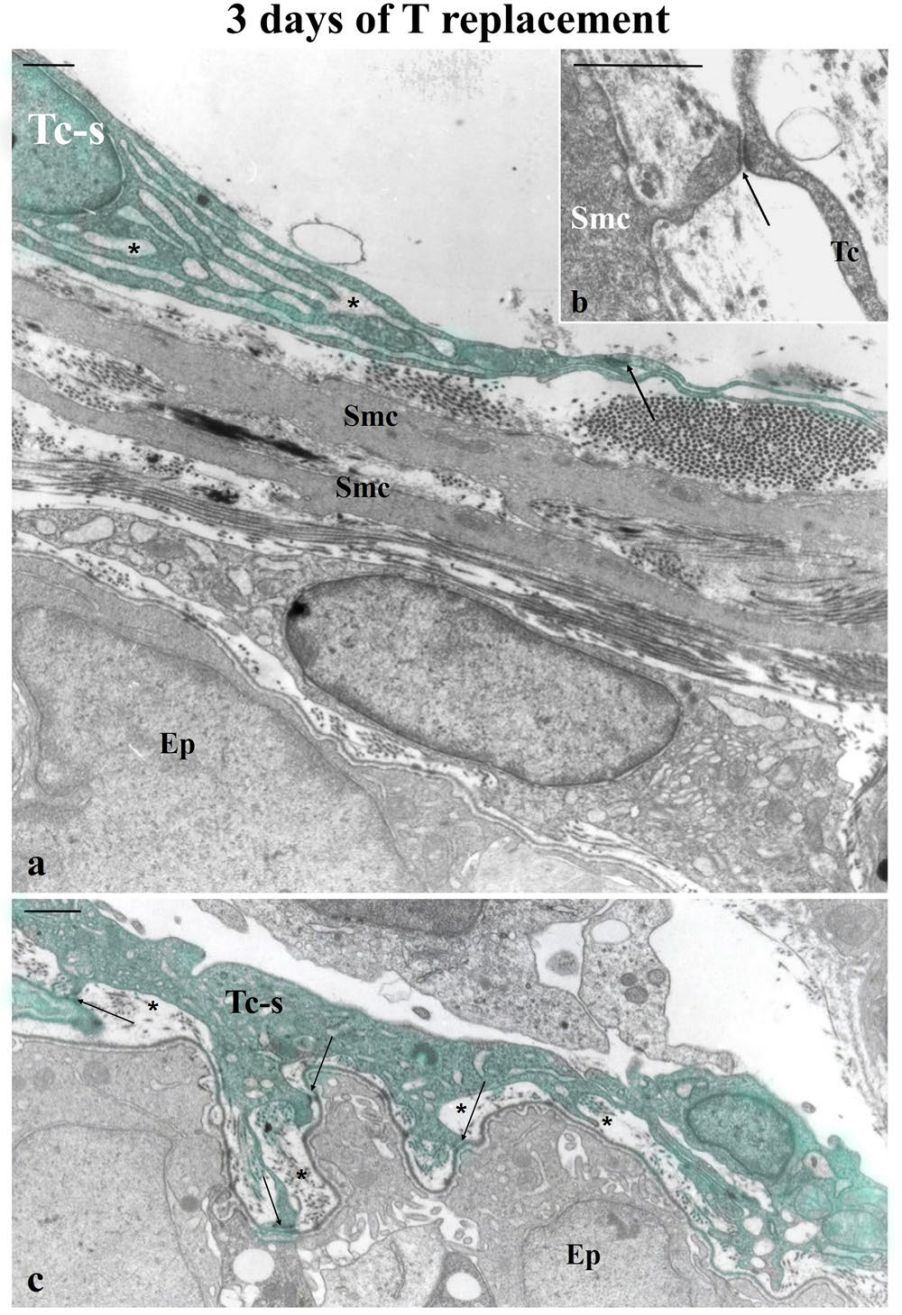
Ultrastructure of the ventral prostate of rats 21 days after castration administered 3 days of T replacement. (**a**) T replacement induced a marked activation of perimuscular telocytes, and these cells exhibited large cytoplasm filled with enlarged cisternae of the rough endoplasmic reticulum (asterisks). Smooth muscle cells appeared at the transition from a synthetic to contractile phenotype with a distended morphology.(**b**) Perimuscular telocytes had focal adhesions with smooth muscle cells. (**c**) Subepithelial telocytes in their activated phenotypes have several focal adhesions (arrows) with the epithelial basement membrane and formed small spaces (asterisks) that contained fine bundles of collagen fibrils, findings that indicated an intimate association between the telocytes and the prostatic epithelium. Abbreviations: Smc (smooth muscle cells); Tc (telocytes); Ep (epithelium). Scale bar is 1 μm.

There was marked activation of the subepithelial and perimuscular prostatic telocytes in the castrated animals after 3 days of testosterone replacement. Specifically, these telocytes demonstrated loose chromatin and a large cytoplasm filled with enlarged cisternae of rough endoplasmic reticulum and the Golgi apparatus, changes that indicated a synthetic phenotype. Smooth muscle cells, with a distended morphology, appeared at the transition from a synthetic to a contractile phenotype (Fig. 4a). Cellular junctions were observed between the telocytes and the smooth muscle cells (Fig. 4b) and also between telocytes and epithelial basement membrane and formed small spaces that contained thin bundles of collagen fibrils, a result that possibly indicates the secretory activity of telocytes (Fig. 4c). Thus, prostatic telocytes were intimately connected with both epithelial and muscle cells when both layers were progressively reorganising, a finding that may indicate an organisational role for telocytes in glandular regrowth.

**Figure 5:**
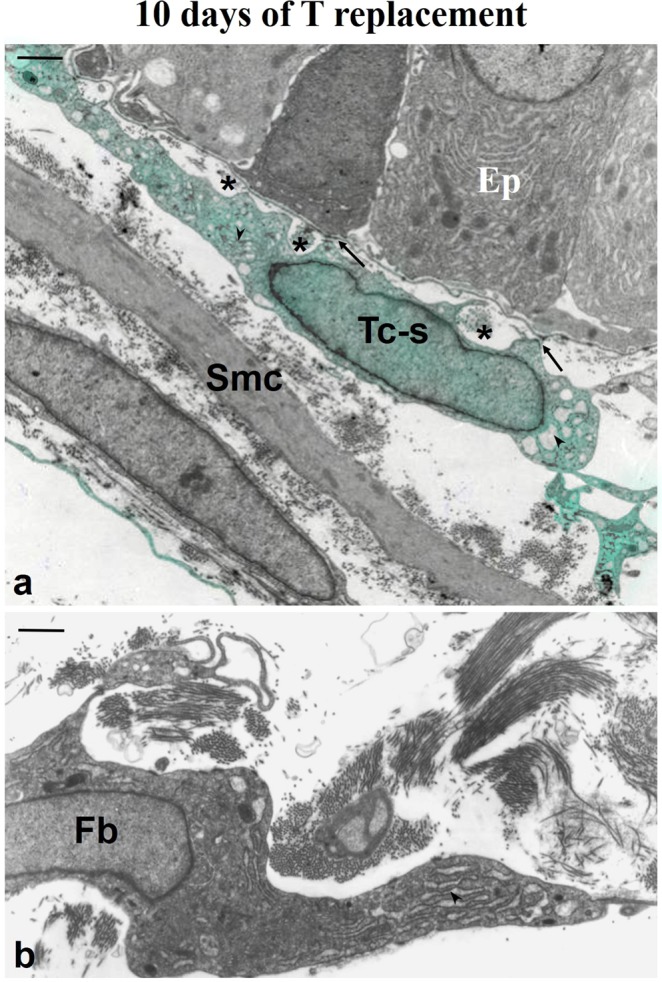
Ultrastructure of the ventral prostate of animals 21 days after castration administered 10 days of T replacement. (**a**) Both subepithelial and perimuscular telocytes exhibited loose chromatin and evident cisternae of rough endoplasmic reticulum and the Golgi apparatus (arrowheads). Subepithelial telocytes also have several focal adhesions (arrows) with the epithelial basal membrane and formed small spaces (asterisks) that contained fine bundles of collagen fibrils (asterisks). Telocyte morphology progressively returned to the normal, non-synthetic phenotype, in which fusiform cell bodies and less thick and unique telopodes occur. (**b**) Detail of an activated interstitial fibroblast that exhibits loose chromatin and evident cisternae of the rough endoplasmic reticulum (arrowheads), and presents a distinct morphology from telocytes with normal or synthetic phenotypes. Abbreviation: Tc-s (telocytes with a synthetic phenotype); Tp (telopodes); Ep (epithelium); Smc (smooth muscle cells); Tp (telopodes). Scale bar is 1 μm.

After 10 days of TR, the subepithelial and perimuscular prostate telocytes exhibited loose chromatin and increased cisternae of rough endoplasmic reticulum and the Golgi apparatus. Subepithelial telocytes also made a sequence of several focal adhesions with the epithelial basement membrane and formed small spaces that contained fine bundles of collagen fibrils. Telocyte morphology progressively returned to the normal, non-synthetic phenotype, where fusiform cell bodies and less thick telopodes occur (Fig. 5a). Activated fibroblasts are also seen, and they have a distinct morphology from normal and synthetic telocyte phenotypes (Fig. 5b).

### Immunohistochemical assays

**Figure 6:**
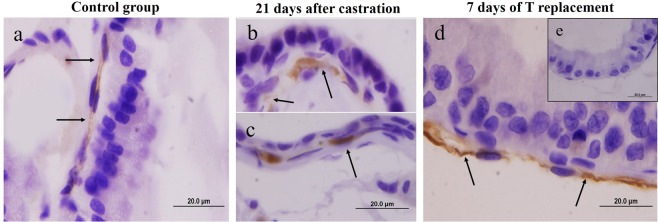
CD34 immunohistochemistry in the ventral prostate from control rats, 21 days after castration and 7 days after T replacement (**a**) In the control group, CD34 immunolabelling was verified in the subepithelial region. The thin cytoplasmic extensions of the telocytes, the telopodes, were CD34 positive. (**b**,**c**) Twenty-one days after castration, another CD34 immunolabelling pattern was observed in the subepithelial region. Immunolabelled cells had abundant cytoplasm, which diverged from typical telocyte immunolabelling. These cells also did not present telopodes. (**c**) After 7 days of T replacement, intense CD34 immunolabelling in the subepithelial region was observed; this staining follows the characteristic pattern of telocytes with CD34 positive telopodes. The arrow indicates CD34 immunolabelling.

Telocytes are CD34-positive cells. Thus, CD34 immunohistochemistry was performed in the VP to localize these cells. In CT animals, CD34 positive cells were localize in subepithelial region; the telopodes were immunolabelled (Fig. 6a). Three and 21 days after castration, CD34 immunostaining was observed in the subepithelial region and these cells exhibited abundant cytoplasmic immunostaining, but CD34 fibrillar-like sections typical of the telopodes are absent (Fig. 6b,c). After 7 days of T replacement, intense CD34 labelling in the subepithelial region revealed the characteristic pattern of telocytes with CD34 positive fibrillar-like sections that are typical of the telopodes (Fig. 6d). The negative control shows no immunostaining (Fig. 6e).

**Figure 7:**
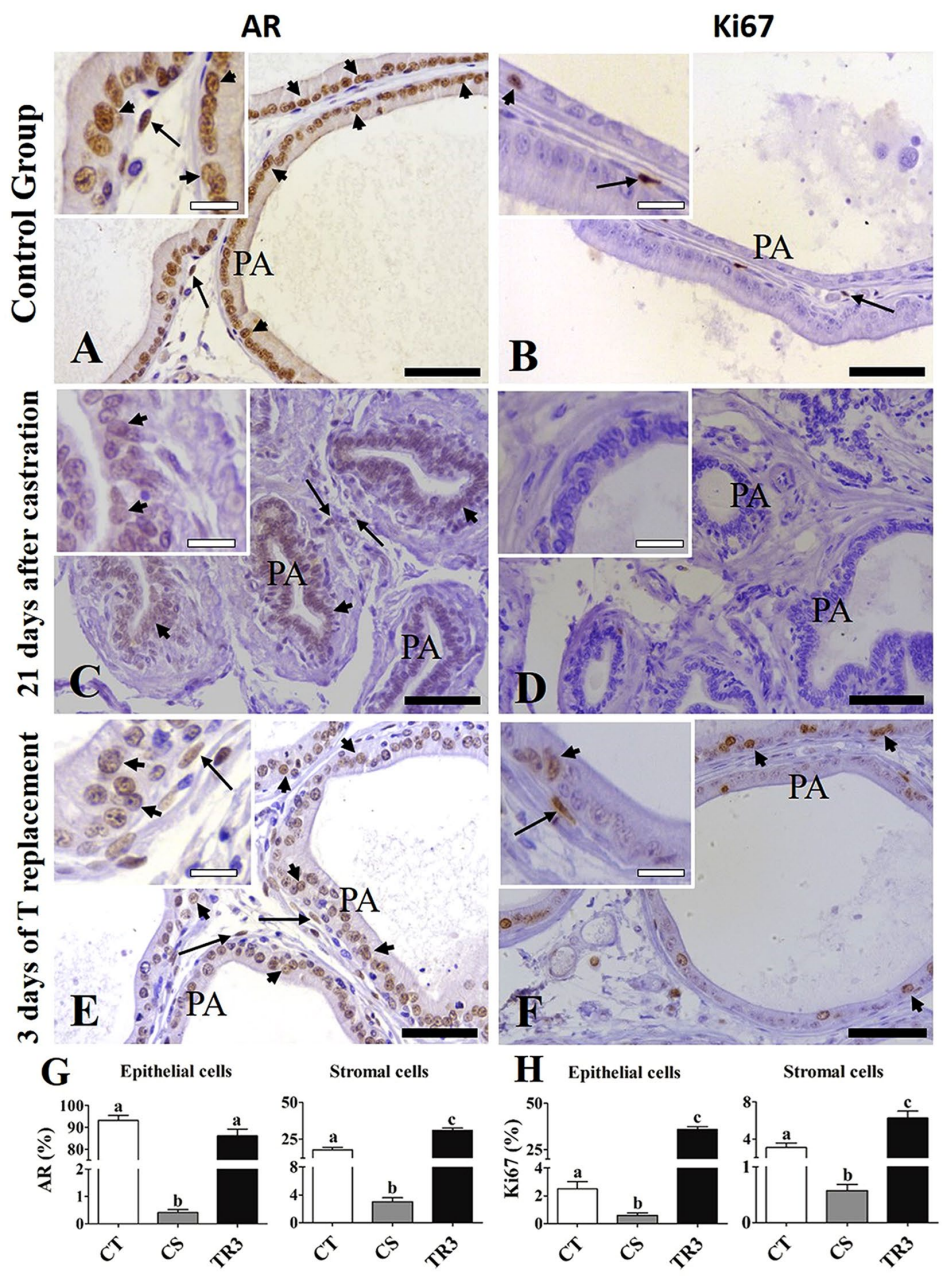
AR and Ki67 immunohistochemistry in the rat ventral prostate from CT, CS21 and TR3 groups. (**A**) In the CT group, strong immunolabeling for AR was observed in the prostatic epithelium and in stromal cells, including in the perialveolar region, in which telocytes are normally detected. (**B**) The immunolabeling for Ki67 is verified on dispersed epithelial and stromal cells. (**C**) Twenty-one days after castration, the immunolabeling for the AR becomes weak in the epithelium, AR-positive stromal cells are observed. (**D**) The immunolabeling for Ki67 becomes reduced in both prostatic epithelium and stroma. (**E**) After 3 days of T replacement, the immunolabeling for AR becomes intense in the prostatic epithelium and AR positive cells are verified in the prostatic stroma, including in the perialveolar region. (**F**) Immunolabeling for Ki67 is verified in both stromal and prostatic epithelial cells, indicating increased proliferative activity. (**H**) The percentage of AR positive cells decreased in both prostatic epithelium and stroma in the CS compared to CT group, but increased in TR3 compared to the CS group. (**G**) The percentage of Ki67 positive cells decreased in both the epithelium and stroma in the CS group compared to the CT, but increased in the TR3 compared to the other two groups. Arrow (stromal immunolabeling), arrowhead (epithelial immunolabeling), PA (prostatic alveoli), bar (50 μm).

In order to evaluate whether castration and hormone replacement had effects on the presence of AR (androgen receptor) and Ki67 immunoassays were performed in ventral rat prostate of the control rats, 21 days after castration and 3 days of T replacement. In the control rats, strong immunolabeling for AR is verified in the prostatic epithelium and in stromal cells including some cells with fusiform nucleus in the perialveolar region, in which telocytes are normally detected (Fig. 7A). The immunolabeling for Ki67 is verified on dispersed epithelial and stromal cells (Fig. 7B). Twenty-one days after castration the immunolabeling for the AR becomes weaker in the epithelium, indicating a reduction in testosterone sensitivity and loss of secretory epithelium. AR-positive stromal cells are observed (Fig. 7C). The immunolabeling for Ki67 becomes reduced in both prostatic epithelium and stroma, which indicates a decrease in proliferative activity (Fig. 7D). After 3 days of T replacement, the immunolabeling for AR becomes intense again in the prostatic epithelium and AR positive cells are verified in the prostatic stroma, including in the perialveolar region (Fig. 7E). Immunolabeling for Ki67 are verified in both stromal and prostatic epithelial cells, indicating increased proliferative activity (Fig. 7F). The percentage of AR positive cells decreased in both prostatic epithelium and stroma in the group of rats analysed 21 days after castration (CS) compared to the control group (CT), but increased in the group of rats analysed after three days of T replacement (TR3) compared to the CS group (Fig. 7H). The percentage of Ki67 positive cells decreased in both the epithelium and stroma in the CS group compared to the CT, but increased in the TR3 group compared to the other two groups (Fig. 7G).

### Immunofluorescence assays

**Figure 8:**
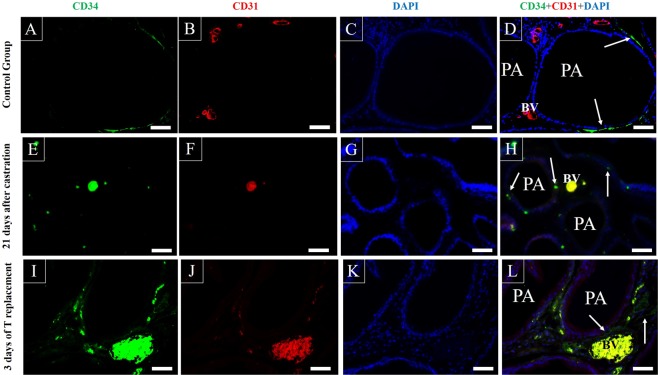
Double immunofluorescence assays for CD34 and CD31 in the ventral prostate of rats of the control group, the group of 21 after castration and the castrated group submitted to 3 days of testosterone replacement. (**A–D**) In the control group, the presence of CD34 immunolabelling in the subepithelial region is verified. The thin cytoplasmic extensions of the telocytes, the telopodia, are marked without the occurrence of colocalization with the CD31. (**E–H**) In the group analysed 21 days after castration, another pattern of CD34 immunolabelling is observed in the subepithelial region, in this region immunolabelled cells with abundant cytoplasm are observed, which diverge from the typical immunolabelling pattern associated with the telocytes. These cells also do not present telopodes and the colocalization with CD31 does not occur in them. The colocalization of CD34 with CD31 is observed in blood vessels and hematopoietic cells. (**I–L**) After 3 days of testosterone replacement, CD34 immunolabeling can be observed in the subepithelial region, with incipient fibrillar-like labeling, which indicates the return of the characteristic pattern of telocytes with the presence of telopodes. The colocalization of CD34 and CD31 is observed in blood vessels and hematopoietic cells. Arrow (CD34 immunolabeling associated with telocytes). PA (Prostatic alveoli), BV (Blood vessels), White bar (100 μm), Yellow bar (50 μm).

In order to distinguish telocytes from other CD34 positive cells, as endothelial and hematopoietic cells, double immunofluorescence assays for CD34 and CD31, that labels endothelial and hematopoietic cells but not the telocytes, were performed. The CD34 positive cells were verified at the periphery of the prostatic alveoli in the control group, these cells have long cytoplasmic projections and are CD31 negative, which coincides with the profile of the telocytes (Fig. 8A–D). In the group analysed 21 days after castration, another pattern of CD34 immunolabeling is observed in the subepithelial region, in this region immunolabelled cells with abundant cytoplasm are observed, which diverge from the typical immunolabeling pattern associated with the telocytes. These cells also do not present telopodes and the colocalization with CD31 does not occur in them. The colocalization of CD34 with CD31 is observed in blood vessels and hematopoietic cells (8e-h). After 3 days of testosterone replacement, CD34 immunolabeling can be observed in the subepithelial region, with incipient fibrillar-like labeling, which indicates the return of the characteristic pattern of telocytes with the presence of telopodes. The colocalization of CD34 and CD31 is observed in blood vessels and hematopoietic cells but not in telocytes (8i-l).

### Serological data

**Figure 9:**
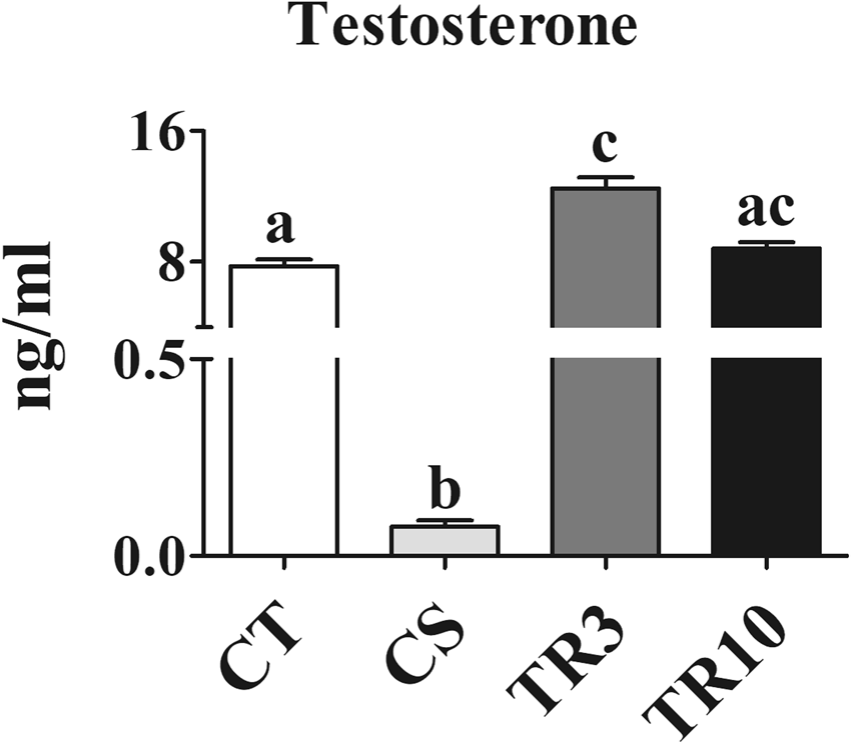
Graph of testosterone serum concentration measured for animals of the Control (CT), after 21 days of castration (CS), after 3 or 10 days of testosterone replacement (TR3 or TR10, respectivelly). Serum levels of testosterone in CS group are below to the detection limit of the test used (0.02 ng/mL). In castrated animals that received T replacement, a higher level was observed in the group analysed after 3 days than that analysed after 10 days of T replacement. The level of serum testosterone of the TR10 group reduces to the CT levels, but the value is not statisticaly significant of TR3 group. Different letters mean statisticaly difference with p < 0.05.

The serum testosterone was 7.1 ± 1.6 ng/mL in CT group. The T levels was below the detection limit (0.02 ng/mL) in CS21 group (Fig. 9). Testosterone levels were higher in rats TR3 group (13.4 ± 1.8 ng/mL) compared to TR10 (9.6 ± 1.3 ng/mL). In TR10 group testosterone level is the same observed in the CT (Fig. 9).

**Figure 10:**
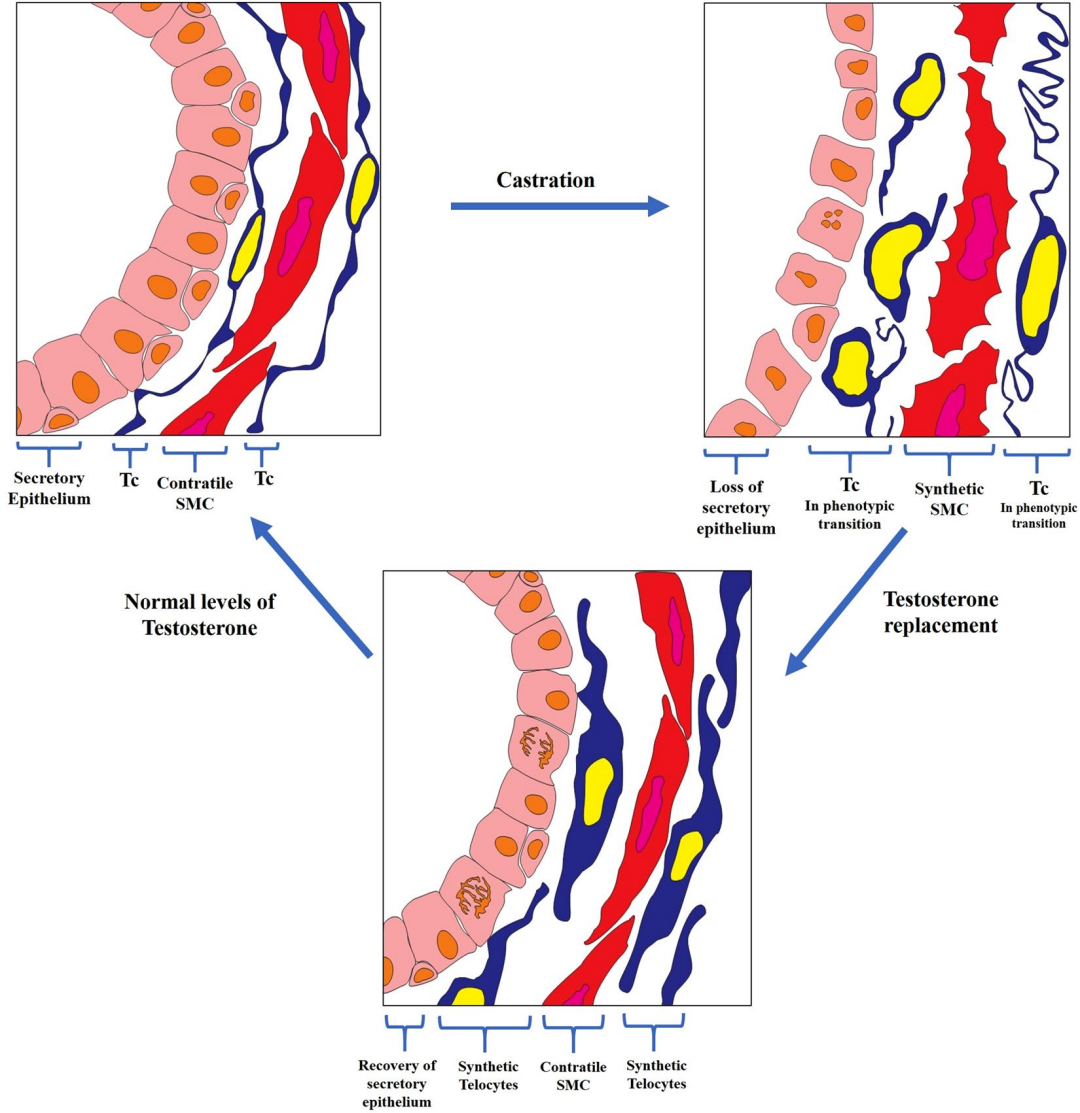
Schematic draw that depicts the modifications to the epithelium, smooth muscle cells and telocytes due to castration and testosterone replacement. In the control group, the telocytes, smooth muscle cells and secretory epithelium exhibit their characteristic morphology. After castration, luminal epithelial cells undergo apoptosis, prostatic epithelium is dominated by basal cells, and subepithelial telocytes exhibit either partial or total loss of telopodes, while perimuscular telocytes show folded telopodes and smooth muscle cells switch to the synthetic phenotype. As testosterone replacement occurs, the secretory epithelium develops again, telocytes exhibit a synthetic phenotype, with both dilated nuclei and cytoplasm and the loss of their telopodes and smooth muscle cells progressively return to their contractile phenotype. Finally, after 10 days of testosterone replacement, as serum testosterone levels return to that seen in the control group, the prostatic secretory epithelium can be observed, smooth muscle cells are in their contractile phenotype and form a thin layer surrounded by telocytes, these progressively return to their characteristic phenotype, with telopodes and a fusiform cell body.

Glandular and cellular changes in epithelium, smooth muscle cells and telocytes due to castration and hormone replacement were presented as a schematic draw in the Fig. 10. Telocytes exhibit remarkable phenotype plasticity under androgen handling.

## Discussion

Castration has drastic effects on the prostate, leading to gland involution, as reflected in the loss of the secretory epithelium and the disorganisation of the supportive prostatic stroma^34,35,38^. Our data corroborate the effects of castration on the prostate and add complexity to this process by implicating a new player: telocytes. According to our ultrastructural and immunohistochemical results, these cells modify their phenotype after castration. Subepithelial telocytes show reduction or loss of their telopodes as well as nuclei dilation. These changes occur simultaneously with the progressive loss of focal adhesions with the disorganised secretory epithelium, indicanting a possible reciprocal interaction between telocytes and epithelial cells for maintenance the normal cellular phenotype.

Smooth muscle cells in the prostate also establish adherens junctions with telocytes. Junctions between these cells were observed in the ileum^39^ and other organs^25^. These junctions are lost over the duration of castration, and smooth muscle cells undergo drastic changes as demonstrated by the acquisition of a synthetic phenotype marked by an irregular shape and associations with bundles of collagen fibres. Telocytes may play a broader role in adult prostate tissue organisation, not only by compartmentalising the stromal microenvironment^26^, but by also actively acting on the secretory epithelium and the smooth muscle layer. However, it should be noted that telocytes are also altered reciprocally by these other tissue components and prostate function maintenance depends on these multidimensional interactions.

Prostate involution reversal was observed by means of T replacement^35^. Three days after T replacement, telocytes acquired a phenotype similar to the smooth muscle cell synthetic phenotype, with dilated cytoplasm, large nuclei and association with bundles of collagen fibrils, and focal adhesions with the epithelium basement membrane and adherens junctions with smooth muscle cells were restored. We term these cells “telocytes with a synthetic phenotype”. After 10 days T replacement, the telocytes progressively lost this phenotype and switched to the normal phenotype, and simultaneously the prostatic secretory epithelium and the smooth muscle cell layer were restored. Serum testosterone levels after 3 days of T replacement were higher than compared to 10 days of T replacement, and thus prostatic secretory epithelium restoration depends on the restoration of serum testosterone to that observed in the control group. As part of this restoration, the smooth muscle cells returned to the contractile phenotype as telocytes progressively reverted to their normal phenotypes. Our immunohistochemical data show that telocytes are AR positive and thus sensitive to testosterone, as previously observed by our group^26^, this possibly corroborates the role of this cell type in the reversion of prostate involution induced by castration. Previous data indicated that telocytes are also sensitive to estradiol^26^, and thus prostate telocytes respond to steroid hormones.

In general, our data suggest that telocytes participate in both maintenance and reestablishment of the secretory epithelium and periductal/perialveolar musculature layer of the prostate after T replacement in castrated animals.

## Conclusion

Our data indicated the presence of telocytes and changes in their morphology during castration-induced prostate involution and prostate involution reversal due to T replacement. We observed morphological changes in telocytes during these processes, with the reduction or absence of telopodes and dilated nuclei during castration and the acquisition of a phenotype, which we termed synthetic, during the onset of T replacement. Finally, after 10 days of T replacement, the telocytes began to return to their characteristic phenotype, which occurred along with the progressive return of T serum levels to the control group level. Finally, our data demonstrated that prostatic telocytes are not static but rather change their phenotypes, and these cells are connected with both the epithelium and smooth muscle and are involved in a T-dependent mechanism that is important both in maintaining the functionality of the prostate and in reversing its involution.

## Material and Methods

### Animals

The animals were provided by the São Paulo State University (UNESP, Botucatu). Adult male 3-months-old Wistar rats were housed in a temperature-controlled (25 °C) room on a 12 h light/dark cycle. All of the animals were housed in polyethylene cages, with ad libitum access to filtered water and rodent food. The experimental protocol followed the Ethical Principles in Animal Research and the Brazilian legislation established by the Brazilian Council of Control in Animal Experimentation and was approved by the Ethics Committee for Animal Use from the Institute of Biosciences of Botucatu/UNESP (CEUA protocol 492). The animals were divided into three groups: Control rats (CT), untreated castrated rats (CS), and castrated rats treated with testosterone replacement (TR).

Castration was performed via orchiectomy at 90 days of age the anesthesy with 100 mL of ketamine (0.116 g mL) and 30 mL xylazine (0.02gmL) (1 mL applied per kg body weight). After 21 days of glandular involution, a group of castrated animals started to receive daily doses of testosterone propionate (4 mg/kg of body weight/day) (Sigma-CO) dissolved in corn oil vehicle, subcutaneous injected, for 10 days. CT and CS animals received only the vehicle.

The animals from CS group were euthanised by lethal injection containing a mixture of an anaesthetic, ketamine (100 mg/kg bw, Dopalen, Vetbrands, Brazil), and a muscle relaxant, xylazine (11 mg/kg bw, Rompun, Bayer, Brazil) after 3 and 21 days of castration, while TR group were killed after 3 and 10 days of the testosterone replacement (8 animals per period of treatment). The ventral prostate (VP) were excised, weighted and immediately immersed in fixative.

### Hormone assay

Blood samples from each animal were collected at the time of death. The plasma was separated and stored at −80 °C. Plasma testosterone (T) concentrations were determined by means of automatic equipment (VITROS ECi-ECiQ analyzer, Johnson & Johnson Inc., New Brunswick, NJ, USA) using specific reagents supplied by Johnson & Johnson Ortho-Clinical (Johnson & Johnson Orthoclinical^TM^, New Brunswick, NJ, USA). The sensitivity of this assay was 0.02 ng/mL. The intra-assay and inter-assay variations for the T assay were 5.36% and 5.10%.

### Immunohistochemistry

Immunohistochemical assays of paraffin-embedded tissues sections were performed using the protocol described in Justulin *et al*.^33^. Ventral prostates were fixed in 4% paraformaldehyde dissolved in phosphate buffer saline for 24 hours. Fixed samples were dehydrated in graded ethanol series, clarified in xylene and embedded in Paraplast (Sigma-CO). Five-µm prostate sections were cut and mounted on silanized slides. Prior to staining, the sections were dewaxed, rehydrated and then unmasked in 0.01 M citrate buffer (pH 6.0). The anti-CD34 (mouse polyclonal, sc74499, Santa Cruz Biotechnology), anti-Ki67 (rabbit monoclonal, ab16667, Abcam), anti-androgen receptor (rabbit polyclonal, sc816, Santa Cruz Biotechnology) antibodies were applied to the sections and incubated overnight at 4 °C. A secondary antibody complexed with peroxidase was utilized and the detection with 3,3′-diaminobenzidine as substrate was done. Counterstaining was performed with Harris hematoxylin. Analyzes were carried out using a DMLB Leica Microscope and the images were obtained by a Leica DFC300FX digital camera connected to the microscope.

### Immunofluorescence assays

Immunofluorescences of paraffin-embedded tissues sections were performed using the protocol described in in Sanches *et al*.^26^. The prostate glands of rats of the control group, the group of 21 after castration and the castrated group submitted to 3 days of testosterone replacement were fixed by immersion in 4% paraformaldehyde (buffered in 0.1 M phosphate, pH 7.4) for 24 h. After fixation, the tissues were washed in water, dehydrated in ethanol series, paraffin embedded (Histosec, Merck, Darmstadt, Germany) and sectioned at 5 μm on a Leica microtome (Leica RM2155, Nussloch, Germany). Such sections of prostate gland were subjected to immunofluorescence assays to evaluate the presence of telocytes throughout the castration induced tissue remodelling and to distinguish these cells from other CD34 positive cells. Therefore, double-immunofluorescence assays for CD34 (mouse polyclonal, IgG, B-6, sc74499, Santa Cruz Biotechnology, Dallas, TX, USA) and CD31(rabbit polyclonal, ab28364, Abcam, Cambridge, MA, USA), a marker of endothelial and hematopoietic cells, were performed; these antibodies were incubated at a dilution of 1:100 overnight. On the following morning, the sections were incubated with goat anti-mouse antibody (FITC, sc-2011, Santa Cruz Biotechnology, Dallas, TX, USA) and goat anti-rabbit (Texas Red, sc-2780, Santa Cruz Biotechnology, Dallas, TX, USA) secondary antibodies diluted 1:200 in 1% BSA for 2 h at room temperature and then stained with DAPI (F36924, Life Technology, Grand Island, NY, USA). Histological sections were analysed with a ZeissImager M2 fluorescence microscope (Zeiss, Germany) coupled with AxioVision software (Zeiss, Germany).

### Transmission electron microscopy (TEM)

Fragments of VP from different experimental groups were processed for TEM. Tissue fragments (1 mm3) were fixed with 3% glutaraldehyde plus 0.25% tannic acid in Millonig’s buffer containing 0.54% glucose^26^. Tannic acid was chosen for better preservation and visualization of the fibrilar components. After washing with the same buffer, the material was post-fixed with 1% osmium tetroxide, washed again, dehydrated in graded acetone, and embedded in Epon 812. Ultrathin silver sections were cut using a diamond knife on a LKB ultramicrotome and contrasted with alcoholic uranyl acetate and lead citrate. Grids were examined in a Phillips CM 100 transmission electron microscope operating at 80 kV.

### Statistical analysis

The values are expressed as mean ± SD. One-way analysis of variance was performed to determine whether differences existed among all groups and then the Tukey Kramer post-test was employed to determine the significance of the differences. A p-value of < 0.05 was considered significant. Statistical analyses were performed using Instat version 3.0 software (GraphPad, San Diego, CA, USA).
